# Multimodal deep learning for acute myocardial infarction detection from 12-lead electrocardiogram: a multi-centre study with cross-hospital validation

**DOI:** 10.1093/ehjdh/ztaf125

**Published:** 2025-10-27

**Authors:** Vibha Gupta, Lukas Hilgendorf, Erik Andersson, Antros Louca, Arman Shahmari, Alfred Hjalmarsson, Rajkumar Saini, Carlo Pirazzi, Monér Alchay, Araz Rawshani

**Affiliations:** Department of Molecular and Clinical Medicine, Institute of Medicine, University of Gothenburg, Bruna Stråket 16, 413 45 Göteborg, Gothenburg, Sweden; Wallenberg Center for Molecular and Translational Medicine, Wallenberg Laboratory, Gothenburg University, Sahlgrenska University Hospital, Blå Stråket 5, Staircase H, 5 B Wallenberglab/SU, Gothenburg 413 45, Sweden; Department of Molecular and Clinical Medicine, Institute of Medicine, University of Gothenburg, Bruna Stråket 16, 413 45 Göteborg, Gothenburg, Sweden; Department of Molecular and Clinical Medicine, Institute of Medicine, University of Gothenburg, Bruna Stråket 16, 413 45 Göteborg, Gothenburg, Sweden; Wallenberg Center for Molecular and Translational Medicine, Wallenberg Laboratory, Gothenburg University, Sahlgrenska University Hospital, Blå Stråket 5, Staircase H, 5 B Wallenberglab/SU, Gothenburg 413 45, Sweden; Department of Molecular and Clinical Medicine, Institute of Medicine, University of Gothenburg, Bruna Stråket 16, 413 45 Göteborg, Gothenburg, Sweden; Wallenberg Center for Molecular and Translational Medicine, Wallenberg Laboratory, Gothenburg University, Sahlgrenska University Hospital, Blå Stråket 5, Staircase H, 5 B Wallenberglab/SU, Gothenburg 413 45, Sweden; Department of Molecular and Clinical Medicine, Institute of Medicine, University of Gothenburg, Bruna Stråket 16, 413 45 Göteborg, Gothenburg, Sweden; Department of Molecular and Clinical Medicine, Institute of Medicine, University of Gothenburg, Bruna Stråket 16, 413 45 Göteborg, Gothenburg, Sweden; Department of Computer Science, Electrical and Space Engineering, Luleå University of Technology, Luleå SE-971 87, Sweden; Department of Thoracic Surgery and Cardiology, Sahlgrenska University Hospital, Blå Stråket 5, Gothenburg, Västra Götaland SE-413 45, Sweden; Department of Molecular and Clinical Medicine, Institute of Medicine, University of Gothenburg, Bruna Stråket 16, 413 45 Göteborg, Gothenburg, Sweden; Department of Thoracic Surgery and Cardiology, Sahlgrenska University Hospital, Blå Stråket 5, Gothenburg, Västra Götaland SE-413 45, Sweden; Department of Molecular and Clinical Medicine, Institute of Medicine, University of Gothenburg, Bruna Stråket 16, 413 45 Göteborg, Gothenburg, Sweden; Wallenberg Center for Molecular and Translational Medicine, Wallenberg Laboratory, Gothenburg University, Sahlgrenska University Hospital, Blå Stråket 5, Staircase H, 5 B Wallenberglab/SU, Gothenburg 413 45, Sweden; Department of Thoracic Surgery and Cardiology, Sahlgrenska University Hospital, Blå Stråket 5, Gothenburg, Västra Götaland SE-413 45, Sweden; Centre for Digital Health, Område Digitalisering, Sahlgrenska University Hospital, Blå Stråket 5, Gothenburg SE-413 45, Sweden

**Keywords:** Acute myocardial infarction (AMI), Electrocardiogram (ECG), Deep learning, Multimodal neural network, External validation, Artificial intelligence in cardiology

## Abstract

**Aims:**

Acute myocardial infarction (AMI) remains a leading global cause of mortality, where timely diagnosis is critical to enable early intervention. The 12-lead electrocardiogram (ECG) is a critical tool for AMI detection. While deep learning (DL) models show promise for automated ECG analysis, most prior studies rely on small, curated datasets with limited external validation, limiting their clinical applicability.

**Methods and results:**

We developed a multimodal DL model (Conv-BiLSTM-Attn) integrating convolutional and recurrent neural networks with an attention mechanism. Using a large, multi-centre dataset of 145 656 ECGs from 96 813 patients across three Swedish hospitals. We trained the model to detect AMI using raw 12-lead ECG signals and demographic inputs (age, sex). Model performance was evaluated under two external validation protocols: generalization across hospitals (GAH) and leave-one-hospital-out (LOHO). The model achieved an area under the receiver operating characteristic (AUROC) of 0.848 (95% CI: 0.84–0.86) and an area under the precision-recall (AUPRC) of 0.456, reflecting class imbalance (∼6–10% AMI prevalence) under the GAH protocol. Subgroup AUROCs ranged from 0.79 to 0.92 across age and sex groups. At the Youden-optimized threshold (0.439), the model showed sensitivity of 0.736, specificity of 0.793, negative predictive value of 0.976, and weighted F1-score of 0.837. Under the LOHO protocol, AUROCs ranged from 0.801–0.849. At Youden thresholds (0.34–0.50), sensitivity ranged from 0.671 to 0.776 and specificity from 0.651 to 0.801, confirming generalizability across sites. Conv-BiLSTM-Attn outperformed benchmark models, with Score-CAM highlighting relevant ST–T segments.

**Conclusion:**

This DL model can support accurate and generalizable AMI detection from routine ECGs, with the Conv-BiLSTM-Attn architecture outperforming current benchmark approaches.

## Graphical Abstract

**Graphical Abstract:**
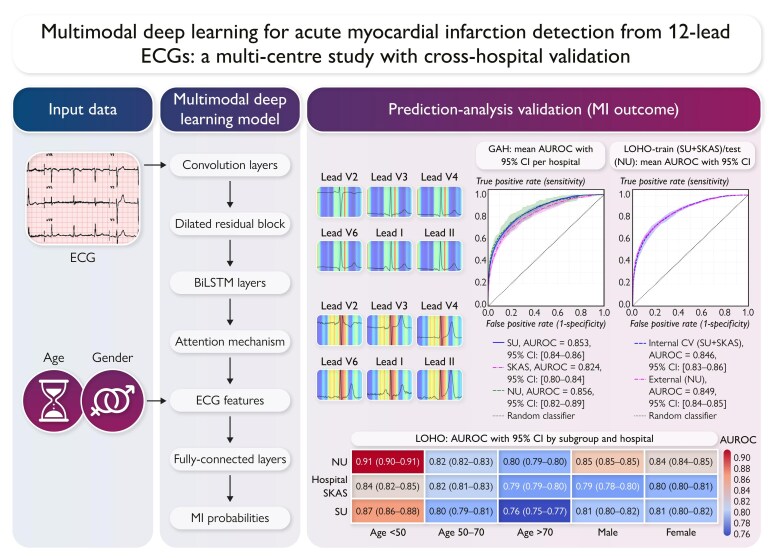

## Introduction

Acute myocardial infarction (AMI) remains a leading cause of morbidity and mortality worldwide. In 2022, cardiovascular diseases (CVDs), including AMI, accounted for approximately 19.8 million deaths globally, about one-third of all deaths.^1,2^ In Europe alone, more than 4 million deaths annually are attributed to CVDs, with myocardial infarction and stroke comprising the majority.^3^

Early and accurate diagnosis of AMI is critical, as timely intervention with reperfusion therapies can significantly reduce myocardial damage and improve patient outcomes.^4,5^ The 12-lead electrocardiogram (ECG) remains the primary diagnostic tool for early detection, particularly in emergency settings where rapid assessment is essential, before cardiac biomarker results are available.^6^ However, accurate ECG interpretation can be challenging due to subtle, non-specific, atypical waveform changes or due to the inexperience of the attending clinician.^7,8^ While ST-elevation myocardial infarction (STEMI) typically produces clear ECG changes, non-ST-elevation myocardial infarction (NSTEMI) often manifests with more subtle alterations, making early detection difficult even for automated approaches.^9^

Deep learning (DL) has demonstrated strong performance in detecting AMI and other cardiac abnormalities using raw 12-lead ECG data.^9–17^ Most prior models, however, relied on unimodal ECG signals and were developed using small or highly curated datasets,^11,18,19^ which may limit generalizability. Incorporating patient demographics, such as age and sex, has been shown to improve diagnostic accuracy in certain cardiac classification tasks.^20,21^

In this study, we developed a multimodal DL model (Conv-BiLSTM-Attn) that combines convolutional and recurrent neural networks with an attention mechanism. Our model leverages a large, multi-centre dataset of real-world ECGs, integrating raw 12-lead signals with demographic information to predict the presence or absence of AMI, aiming for robust performance across diverse emergency department populations.

## Method

### Ethical approval

This study was approved by the Swedish Ethical Review Authority (Dnr 2023-03921-01).

### Study design, data collection, and pre-processing

**Figure 1:**
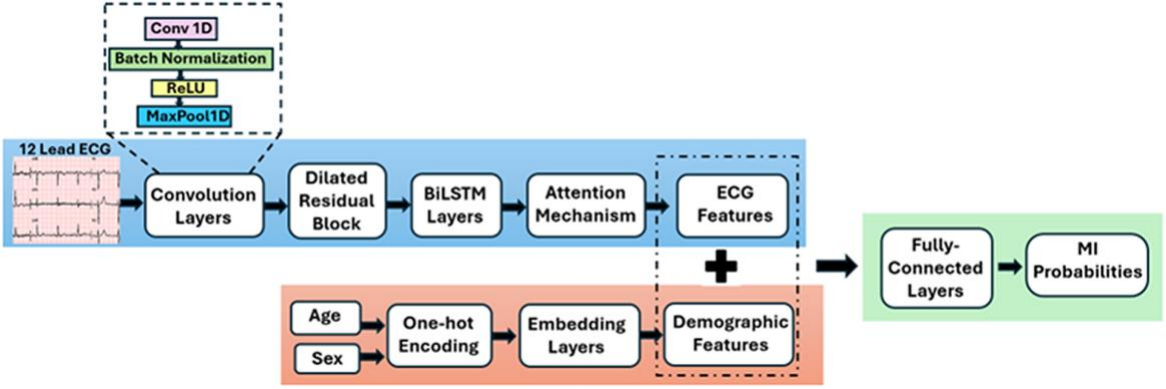
Overview of the proposed Conv-BiLSTM-Attention model architecture for acute myocardial infarction detection. The model combines convolutional layers (with a residual block using dilated convolutions), BiLSTM layers, and an attention mechanism to learn temporal and clinically relevant features from 12-lead ECGs. Age and sex are integrated before final classification.

This retrospective cohort study included adult patients who underwent standard 12-lead electrocardiograms within 6 h of presenting to the emergency departments of three hospitals in the Västra Götaland region of Sweden (*Figure 1*): Sahlgrenska University Hospital (SU), Norra Älvsborgs Länssjukhus (NU), and Skaraborg Hospital (SKAS). The study period spanned from 1 January 2015 to 30 June 2023. ECGs were collected from patients presenting with chest pain or dyspnoea, symptoms suggestive of AMI. Patient labels were assigned based on final hospital discharge diagnoses using ICD-10 codes.^22^ Patients with the code I21 were classified as having experienced an AMI. Patient age and sex were extracted from electronic medical records and incorporated into the analysis to account for demographic variability in AMI presentation.

ECG recordings were originally received in XML format encoded in base64, along with corresponding sampling frequency information. Signals were decoded and stored in WFDB format.^23^ All recordings used a standard 10-s, 12-lead configuration sampled at 500 Hz and subsequently down-sampled to 250 Hz for computational efficiency.^24^

Minimal pre-processing was applied to preserve signal integrity. A first-order Butterworth band-pass filter (0.5–40 Hz, sampling rate = 250 Hz) was applied with zero-phase forward–backward filtering (*filtfilt*) to remove baseline wander and high-frequency noise, a range shown to maintain diagnostically relevant ECG features while improving signal quality.^25–27^ ECG amplitudes were normalized using *Z*-score normalization across leads. All pre-processing steps were applied uniformly using a standardized batch pipeline. Only ECGs with complete demographic information (age and sex) were included in the analysis, and no data imputation was necessary.

### Model architecture (Conv-BiLSTM-Attn)

The proposed convolutional bidirectional-long short-term memory-attention (Conv-BiLSTM-Attn) (*Figure 1*) combines convolutional, recurrent, and attention-based layers to detect AMI from 12-lead ECGs. One-dimensional convolutional layers extract local signal features such as QRS morphology and ST-segment deviations, followed by batch normalization and pooling layers to enhance feature stability and reduce overfitting. Dilated convolutions within a residual block increase the receptive field, allowing for long-range temporal feature extraction.

The extracted representations are processed by bidirectional long short-term memory (BiLSTM) layers, capturing sequential dependencies in both temporal directions. An attention mechanism is applied to emphasize time points most relevant to the prediction task. Patient age and sex are incorporated as auxiliary features by concatenation with the learned ECG representation. The final classification layer outputs AMI probabilities using a SoftMax function. The model was trained using binary cross-entropy loss with label smoothing and optimized using the Adam optimizer. Early stopping was employed to prevent overfitting by monitoring the validation area under the receiver operating characteristic curve (AUROC), halting training when performance plateaued.^28^ Learning rate scheduling was also guided by validation performance. All models were implemented using the TensorFlow (2.10.1) framework^29^ and NVidia GeForce RTX 4090.

### Experimental and training protocol

Model generalizability was assessed using two evaluation protocols: generalization across hospitals (GAH) and leave-one-hospital-out (LOHO). In GAH, five-fold stratified cross-validation was performed on pooled data (aggregated data from all hospitals) from all three hospitals, ensuring proportional representation from each site/hospital in both training and test sets. Model performance was reported using internal validation folds. This setup emulates a real-world deployment where a model encounters new patients from institutions it was trained on.

In LOHO, one hospital was held out entirely for testing while the model was trained on data from the other two hospitals. This was repeated such that each hospital served as an external test site once. Five-fold stratified cross-validation within the training set provided internal validation, while evaluation on the held-out hospital assessed external generalizability across institutional settings.

To reflect clinical practice where patients often have multiple ECG recordings, data splitting was performed at the recording level rather than strictly by patient. While this approach may introduce some dependency between training and test sets, it preserves the natural variability of ECGs encountered in practice. Moreover, strict patient-level splitting in highly imbalanced datasets can reduce sample diversity and negatively impact generalizability. We acknowledge this trade-off and mitigate its impact by robust external validation on independent cohorts.

### External models for benchmarking

To contextualize performance, the Conv-BiLSTM-Attn model was benchmarked against a few established DL architectures: Model A: A ResNet-based model adapted from Han *et al*. (2020),^30^ originally developed for MI detection using the PTB dataset. It uses lead-wise residual blocks with global fusion. Model B: Adapted from Ribeiro *et al*. (2020),^14^ this model was pretrained on over 2 million ECGs from the CODE study for multiclass diagnosis (excluding AMI). We evaluated both a version fine-tuned on our AMI dataset (Model B-TF) and a version trained from scratch. We also included recently published ECG-SMART-NET,^15^ a CNN developed for occlusion myocardial infarction (OMI) detection using angiographically confirmed cases from a narrowly defined cohort. Its evaluation and comparison are further detailed in the Discussion section.

### Evaluation metrics and statistical analysis

Model performance was assessed using the AUROC, area under the precision-recall curve (AUPRC), sensitivity, specificity, and negative predictive value (NPV). Given the imbalanced nature of the dataset (AMI prevalence ranging from 6 to 10% across sites), we report both binary and weighted F1-scores. The binary F1-score focuses on the positive class (AMI), highlighting the model’s ability to correctly identify infarction cases. The weighted F1-score, in contrast, accounts for both classes proportionally based on their frequency, providing a more holistic view of overall performance. Including both metrics ensures that the evaluation reflects both clinical sensitivity and generalizability across all patient presentations. All metrics were reported with 95% confidence intervals (CIs), estimated via bootstrapping. To provide a comprehensive view of model behaviour and support clinical decision-making, two complementary evaluation strategies were employed:

Threshold-dependent performance analysis: To explore how performance metrics varied across different probability thresholds, we computed sensitivity, specificity, NPV, and F1-score (binary and weighted) across a fixed range of thresholds (0.01–0.50). For each threshold, these metrics were averaged across five cross-validation folds and plotted. This analysis was intended to visualize trade-offs and assist in threshold selection, but was not used for final performance reporting.

Youden index-optimized threshold evaluation^31^: To simulate real-world deployment, we selected thresholds that maximized Youden’s *J* statistic (sensitivity + specificity − 1) on each fold. Binary predictions were generated using these optimal thresholds. We then computed bootstrapped estimates (1000 resamples) of mean sensitivity, specificity, NPV, and F1-score (binary and weighted), along with their 95% CIs. These values represent the model’s expected performance under threshold-optimized operation.

Statistical significance was assessed using paired t-tests to compare AUROC values between the proposed Conv-BiLSTM-Attn model and benchmark models (Model A, Model B, Model B-TF, and ECG-SMART-NET) on the same test sets. DeLong’s^32^ test for correlated ROC curves was also applied to evaluate differences in discriminative performance.

Given differences in evaluation protocols, results from the GAH and LOHO setups were analysed independently and not directly compared. Clinical utility was further evaluated using decision curve analysis (DCA),^33^ which quantified the net benefit of each model across a range of risk thresholds relevant to emergency decision-making.

Model interpretability was evaluated using ScoreCAM, a class-discriminative visualization method that highlights the most important time points contributing to the model’s prediction. For each ECG, we extracted the middle heartbeat (the heartbeat located at the centre of the recording) to reduce variability from recording start times. ScoreCAM maps were computed for these middle beats and then averaged across 150 correctly classified positive and 150 negative samples, following the approach used in ECG-SMART-NET. The averaged attention maps were overlaid on individual representative middle beats rather than on averaged ECG signals. All 12 ECG leads were visualized to provide a comprehensive view of the model’s attention patterns.

### Model interpretability

Model interpretability was evaluated using ScoreCAM,^34^ a class-discriminative visualization method that highlights time points contributing most to the model’s prediction. The final temporal convolutional layer was used to generate per-time-point importance scores. For each ECG, we extracted the middle heartbeat (the heartbeat located at the centre of the recording) to reduce variability from recording start times. ScoreCAM maps were computed for these middle beats and then averaged across 150 correctly classified positive and 150 negative samples, following the approach used in ECG-SMART-NET.^15,35^ The resulting average attention maps were overlaid on individual representative middle beats from positive and negative examples, rather than on averaged ECG signals, illustrating the regions and leads the model focuses on for its decisions. All 12 ECG leads were visualized to provide a comprehensive interpretation of the model’s attention patterns. To preserve interpretability, raw ECG signals were not averaged; instead, representative real ECG samples were used to overlay the average ScoreCAM heatmaps, following established methods in clinical AI explainability studies.^36,37^

For visualization, two approaches were used. In the main figures, colour scales were autoscaled within each panel to maximize visibility of variations. In supplementary analyses, examples were plotted with a fixed global colour scale (0–95th percentile of all attention values pooled across positive and negative cases) to allow direct comparability across individual patients.

The heatmaps use the ‘jet’ colormap, where colours transition from blue (indicating minimal model attention), through green, to red (indicating the highest attention), providing an intuitive gradient scale to interpret the importance of different ECG regions. To facilitate transparency and reproducibility, all code is available via GitHub (https://github.com/Vibha190685/ECG-AMI-Detection/tree/main). The complete model architecture and training hyperparameters are summarized in Supplementary material online, *Table S2*.

## Results

A total of 145 656 ECG recordings from 96 813 patients across three hospitals, SU (92 506 ECGs), NU (19 487), and SKAS (30 930), were included in the analysis (see Supplementary material online, *Figure S1*). The mean age of patients was 63.5 years, and 52.1% were male. The prevalence of AMI varied by site/hospital, ranging from 5.9 to 9.6%. ECGs were collected from patients presenting symptoms suggestive of AMI, such as chest pain or dyspnoea. However, only 4.2% of cases had structured symptom annotations. Therefore, symptom data were not used in model development. Detailed demographic and clinical characteristics for each site are summarized in *Table 1*, and the distribution of common comorbidities across hospitals is presented in Supplementary material online, *Table S3*. Training/validation loss curves are provided in Supplementary material online, *Figure S2*.

**Table 1:** Patient demographics and AMI distribution: hospital-wise and age-stratified cohort characteristics, including total ECGs, mean age, sex distribution, overall MI prevalence, and age-specific MI case proportions

Hospital	Total ECGs	Mean Age (±SD)	% Male	AMI prevalence (%)	AMI cases (<50) (%)	AMI cases (50–70) (%)	AMI cases (>70) (%)
SU	92 505	60.5 ± 19.7	51.4	5427(5.9)	350 (1.39)	2226(7.08)	2851(8.39)
SKAS	30 929	72.4 ± 14.8	53.6	2984 (9.6)	96 (3.93)	1017(9.50)	1872(11.58)
NU	19 487	60.7 ± 19.3	53.4	1433(7.4)	74(1.38%)	502 (7.30)	857(11.85)

Values are mean ± SD.

AMI, acute myocardial infarction.

### Model performance under GAH and LOHO protocols

Under the GAH protocol, where data from all three sites were pooled, the Conv-BiLSTM-Attn model achieved strong overall performance, with an AUROC of 0.848 (95% CI: 0.84–0.86) and an AUPRC of 0.456 (95% CI: 0.44–0.47). When broken down by hospital, model discrimination remained stable: NU yielded an AUROC of 0.853 and AUPRC of 0.495, SKAS had an AUROC of 0.822 and AUPRC of 0.456, and SU had an AUROC of 0.851 and AUPRC of 0.446.

**Figure 2:**
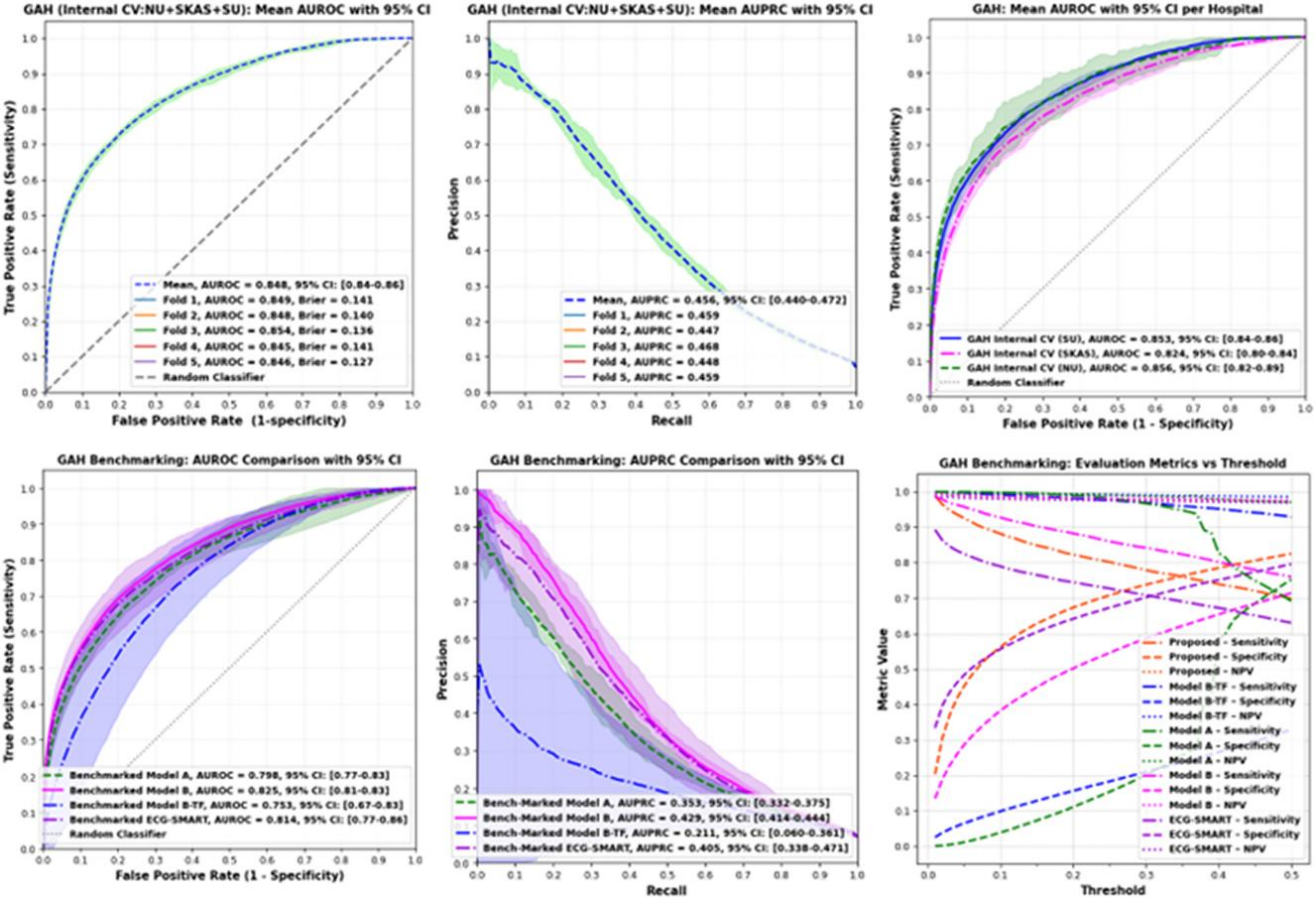
Model performance across generalization across hospitals validation and benchmarking. Top row (left to right): The Conv-BiLSTM-Attn model’s mean receiver operating characteristic curve with 95% confidence interval over five internal cross-validation splits on the pooled generalization across hospitals dataset; the corresponding mean precision–recall curve with 95% confidence interval; and the Conv-BiLSTM-Attn model’s mean receiver operating characteristic curves with 95% confidence interval when tested separately on each hospital (SU, SKAS, NU). Bottom row (left to right): Mean receiver operating characteristic curve with 95% confidence interval for all benchmark models; mean precision–recall curve with 95% confidence interval for the same benchmarks; and threshold-dependent performance analysis (sensitivity, specificity, negative predictive value) for each benchmark model.

**Figure 3:**
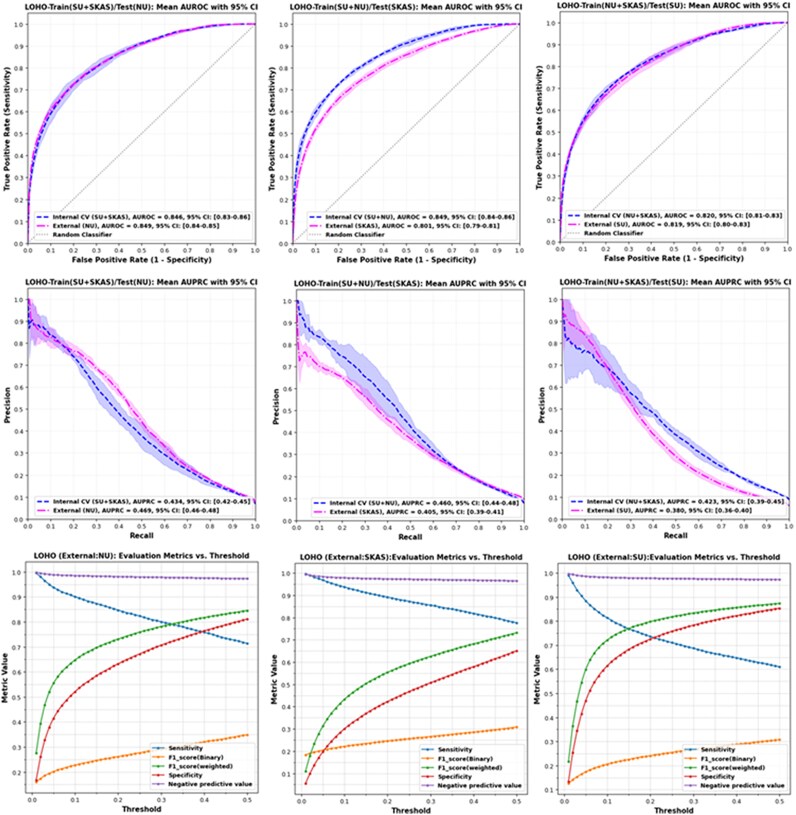
Model performance across leave-one-hospital-out validation and threshold-based sensitivity analysis. Top row: area under the receiver operating characteristic with 95% confidence interval for both internal cross-validation and external validation under three leave-one-hospital-out settings, (left) SU held out, (centre) NU held out, and (right) SKAS held out. Middle row: area under the precision-recall evaluated under the same leave-one-hospital-out configurations, highlighting model performance in the context of class imbalance. Bottom row: Threshold-dependent behaviour analysis for external validation sets from each held-out hospital. Plots illustrate key evaluation metrics, sensitivity, specificity, negative predictive value, and accuracy across a range of decision thresholds, offering insight into optimal operating points and trade-offs relevant to clinical deployment.

In the LOHO setup, where one hospital was excluded for external validation, the model continued to perform well despite domain shifts (i.e. differences in hospital-specific demographics, data acquisition protocols, and patient populations). External validation yielded AUROC values of 0.801 (SKAS), 0.849 (NU), and 0.819 (SU), with corresponding AUPRCs of 0.405, 0.469, and 0.380, respectively. Internal validation within training subsets revealed AUROC values ranging from 0.820 to 0.849, further affirming the model’s generalizability across different institutional contexts. Performance curves and threshold-based metrics for both protocols are shown in *Figures 2* and *3*.

### Threshold-specific diagnostic metrics evaluation

To assess threshold-dependent performance, we evaluated model outputs at the Youden-optimal threshold, which balances sensitivity and specificity by maximizing Youden’s *J* statistic.

Across hospitals in the LOHO protocol, the Youden-optimized thresholds were NU – 0.452, SU – 0.338, and SKAS – 0.500 (see Supplementary material online, *Table S2*). Performance at these thresholds varied modestly across sites: sensitivity ranged from 0.671 to 0.776, specificity from 0.651 to 0.801, and NPV from 0.965 to 0.975. For instance, at NU, the model achieved a sensitivity of 0.737 (95% CI: 0.72–0.77), specificity of 0.789 (95% CI: 0.75–0.81), and NPV of 0.974 (95% CI: 0.97–0.98). Binary F1-scores ranged from 0.276 to 0.337, and weighted F1-scores ranged from 0.731 to 0.844, reflecting the class imbalance.

Performance under the GAH protocol showed similar trends, with a Youden-optimized threshold of 0.439. At this threshold, the model achieved a sensitivity of 0.736 (95% CI: 0.71–0.76), specificity of 0.793 (95% CI: 0.76–0.81), and an NPV of 0.976 (95% CI: 0.97–0.98). The binary and weighted F1-scores were 0.325 (95% CI: 0.31–0.34) and 0.837 (95% CI: 0.82–0.85), respectively.

Full diagnostic results, including performance at Youden-optimized thresholds and exploratory analysis at a fixed low threshold (0.01 for high-NPV rule-out), are available in Supplementary material online, *Table S1*.

### Subgroup performance by age and sex

**Figure 4:**
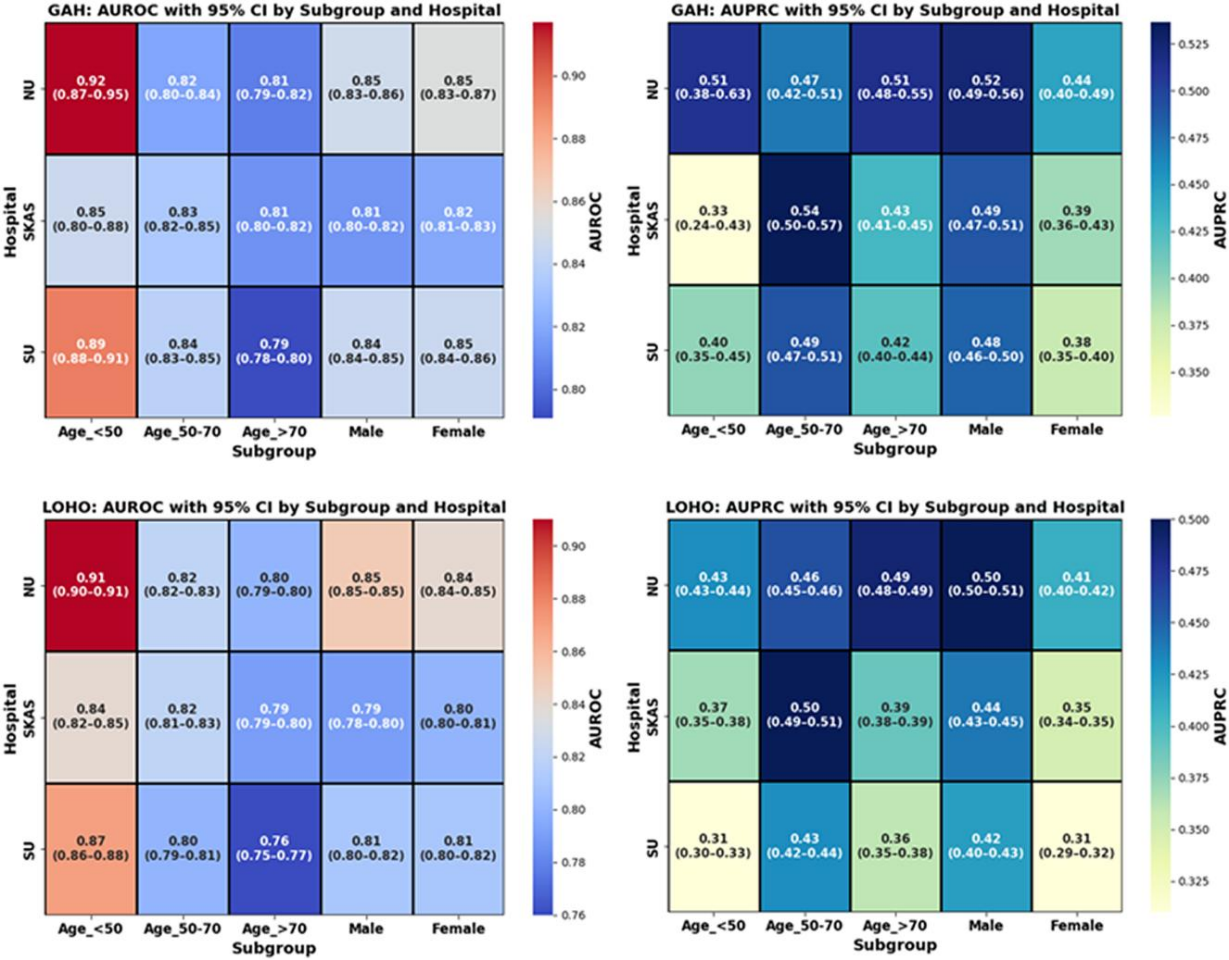
Heatmaps showing the area under the receiver operating characteristic and the area under the precision-recall values by hospital and patient subgroup across the leave-one-hospital-out and generalization across hospitals protocols.

Subgroup analyses were conducted across predefined age categories (<50, 50–70, >70)^38^ and sex. Across all subgroups, the AUROC ranged from 0.761 to 0.920, and AUPRC remained consistently above 0.30, even in low-prevalence strata. Similar patterns were observed under both LOHO and GAH protocols (*Figure 4*).

Subgroup prevalence varied substantially. For example, patients under 50 had an MI prevalence of only ∼1.3%, yet this translated to more than 350 MI-positive cases at SU alone. In contrast, older adults (>70) at NU exhibited a prevalence of 11.8%, with a total of 857 cases. These differences in case count and distribution likely contributed to variation in model performance. Supplementary material online, *Table S1* presents demographic and clinical characteristics of each hospital-specific cohort, including age, sex distribution, and AMI prevalence.

### Comparative benchmarking and statistical validation

Due to the substantial training demands of the LOHO, we primarily used this protocol for comparative benchmarking. Under this setting, the Conv-BiLSTM-Attn model consistently demonstrated superior performance against all benchmark models. It achieved an AUROC of 0.848 (95% CI: 0.84–0.86) and an AUPRC of 0.456 (95% CI: 0.44–0.47), outperforming all competing architectures. The ResNet-based Model A yielded an AUROC of 0.790 (95% CI: 0.77–0.83) and an AUPRC of 0.350 (95% CI: 0.33–0.37), while Model B, pretrained on over 2 million ECGs from the CODE study, reached 0.823 (95% CI: 0.81–0.83) and 0.430 (95% CI: 0.41–0.44), respectively. Model B-TF performed less favourably with an AUROC of 0.750 (95% CI: 0.67–0.83) and AUPRC of 0.210 (95% CI: 0.06–0.36). The ECG-SMART-NET model, which has recently been proposed for ECG-based classification tasks, achieved an AUROC of 0.814 (95% CI: 0.77–0.86) and an AUPRC of 0.405 (95% CI: 0.34–0.47), performing competitively but below the proposed method.

At the Youden-optimal threshold, the Conv-BiLSTM-Attn model offered a well-balanced diagnostic profile with a sensitivity of 0.736 (95% CI: 0.71–0.76), specificity of 0.793 (95% CI: 0.76–0.81), NPV of 0.976 (95% CI: 0.97–0.98), F1 binary score of 0.325 (95% CI: 0.31–0.34), and a weighted F1-score of 0.837 (95% CI: 0.82–0.85). In comparison, Model A achieved a sensitivity of 0.710 (95% CI: 0.67–0.77), specificity of 0.737 (95% CI: 0.68–0.78), and a weighted F1-score of 0.798 (95% CI: 0.77–0.83), indicating comparatively lower discriminative performance. Model B showed slightly improved sensitivity at 0.743 (95% CI: 0.71–0.84), with specificity of 0.711 (95% CI: 0.61–0.78), and a weighted F1 of 0.784 (95% CI: 0.72–0.83). While Model B-TF achieved the highest sensitivity of 0.929 (95% CI: 0.88–0.98), its low specificity of 0.329 (95% CI: 0.17–0.51) and reduced weighted F1-score of 0.454 (95% CI: 0.2876–0.64) undermined its clinical utility. ECG-SMART-NET achieved a sensitivity of 0.721 (95% CI: 0.59–0.87), specificity of 0.731 (95% CI: 0.48–0.86), and a weighted F1-score of 0.789 (95% CI: 0.61–0.87), reflecting a reasonable balance, but still falling short of the proposed model’s overall performance.

DCA, presented in Supplementary material online, *Figure S3*, further confirmed that the Conv-BiLSTM-Attn model provided the highest net clinical benefit across probability thresholds ranging from 5% to 20%.

Statistical comparisons demonstrated that the proposed Conv-BiLSTM-Attn model significantly outperformed all benchmark models. Paired *t*-tests confirmed improvements over Model A (*t* = 83.10, *P* < 0.001), Model B (*t* = 15.20, *P* < 0.001), Model B-TF (*t* = 4.35, *P* = 0.012), and ECG-SMART-NET (*t* = 3.73, *P* = 0.020). In addition, DeLong’s test for correlated ROC curves confirmed significant improvements for all benchmarks (all *P* < 0.001). Performance gains ranged from modest but meaningful improvements over strong pretrained architectures (ΔAUC = 0.02 vs. Model B) to substantial margins over other baselines (ΔAUC = 0.32 vs. Model A; ΔAUC = 0.10 vs. Model B-TF; ΔAUC = 0.07 vs. ECG-SMART-NET).

Statistical comparisons demonstrated that the proposed Conv-BiLSTM-Attn model significantly outperformed all benchmark models. Paired *t*-tests on cross-validation folds showed improvements over Model A (*t* = 83.10, *P* < 0.001), Model B (*t* = 15.20, *P* < 0.001), Model B-TF (*t* = 4.35, *P* = 0.012), and ECG-SMART-NET (*t* = 3.73, *P* = 0.020). In addition, DeLong’s test for correlated ROC curves confirmed significant improvements for all benchmarks (all *P* < 0.001). Performance gains ranged from modest but meaningful improvements over strong pretrained architectures (ΔAUC = 0.02 vs. Model B) to substantial margins over other baselines (ΔAUC = 0.32 vs. Model A; ΔAUC = 0.10 vs. Model B-PR; ΔAUC = 0.07 vs. ECG-SMART-NET).

### Impact of demographic features and visual interpretability

To evaluate the added value of demographic variables, model performance was compared with and without the inclusion of age and sex as input features. Excluding demographics led to a small performance drop: AUROC decreased from 0.848 (95% CI: 0.84–0.84) to 0.842 (95% CI: 0.83–0.85), and AUPRC from 0.456 (95% CI: 0.44–0.47) to 0.455 (95% CI: 0.446–0.47). At the Youden threshold, sensitivity declined slightly from 0.736 to 0.730, while specificity remained unchanged. Inclusion of demographic variables resulted in marginal improvements in model performance, indicating that the ECG waveform itself carries substantial diagnostic information for myocardial infarction prediction.

**Figure 5:**
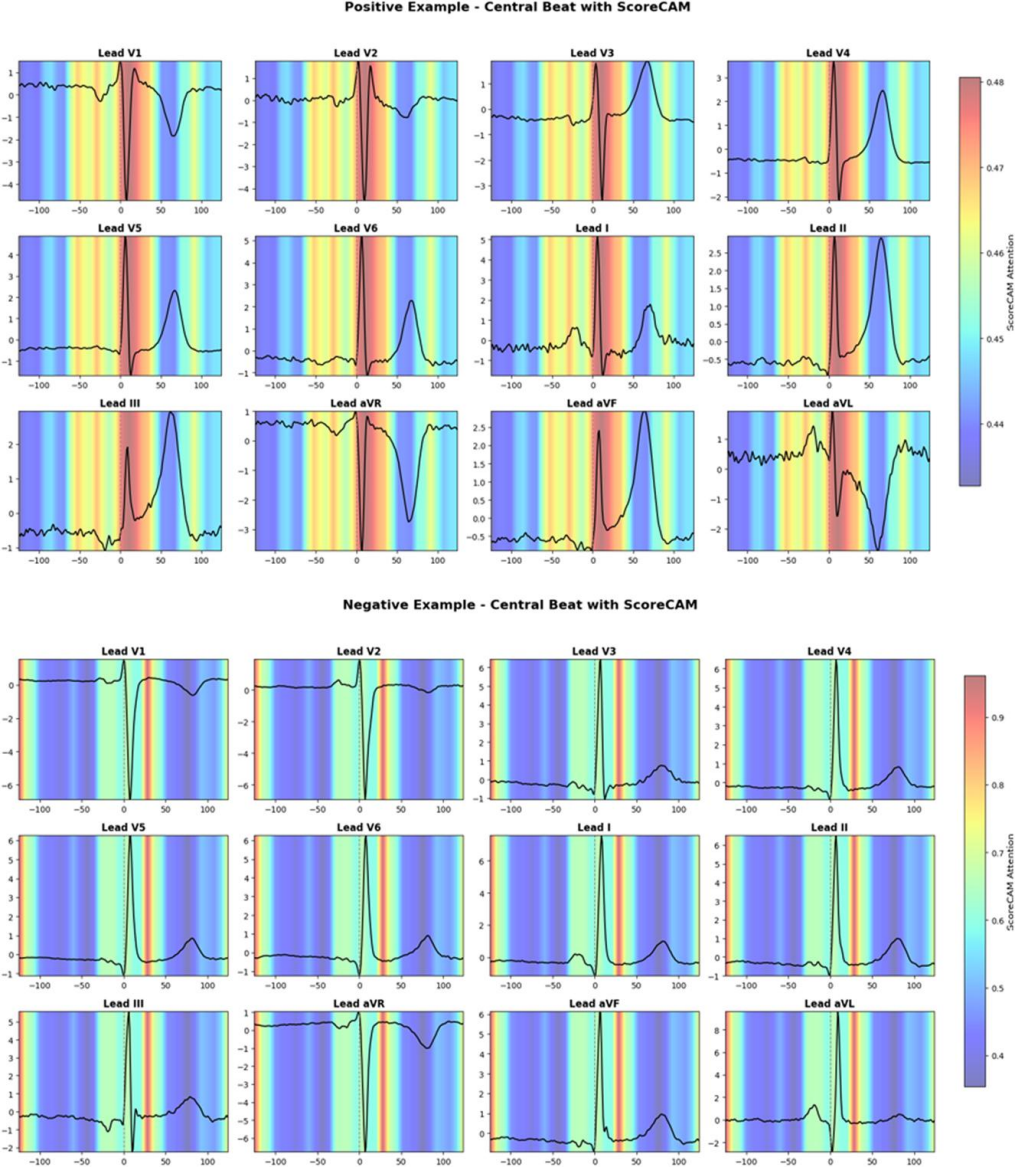
ScoreCAM-based attention maps overlaid on representative ECG signals for correctly classified myocardial infarction-positive (top) and myocardial infarction-negative (bottom) cases. Heatmaps represent the average attention across 150 correctly classified samples per class, displayed on a single representative middle heartbeat. Region with stronger model attention indicate more relevant signal segments, while region with lower attention indicate less relevance. Colour bars are autoscaled for each panel, which compresses the dynamic range in the positive case; for direct comparison across cases, see Supplementary material online, *Figure S4*, where a fixed global scale (0–95th percentile) is applied.

Model interpretability was examined using ScoreCAM visualizations on correctly classified AMI-positive and AMI-negative cases (*Figure 5*). Beat-centred maps showed that attention was frequently concentrated around the QRS complex and ST segment, with consistent activation patterns across representative samples. Warmer colours (yellow/red) indicate regions of higher attention, while cooler colours (blue) indicate less relevant areas.

Because class-mean ScoreCAM maps were averaged across 150 patients, small temporal and morphological variations attenuated peak attention in the positive class, resulting in a narrower apparent colour scale compared with the negative case. This reflects averaging effects rather than inconsistent model behaviour. To ensure comparability, Supplementary material online, *Figure S4* provides additional representative positive and negative cases plotted with a fixed global scale (0–95th percentile of all attention values), confirming that the model consistently highlights physiologically relevant ECG regions associated with myocardial infarction.

## Discussion

In this large, multi-centre, retrospective cohort study, we developed and externally validated a DL model (Conv-BiLSTM-Attn) for automated detection of AMI from standard 12-lead ECGs. Using over 140 000 ECGs from three emergency hospitals with varying patient demographics and MI prevalence, the model demonstrated robust and generalizable performance, achieving AUROCs exceeding 0.82 across individual sites and a pooled AUROC of 0.85. The model maintained strong accuracy under an LOHO validation scheme, underscoring its generalizability and adaptability across diverse clinical settings with varying patient demographics and ECG acquisition protocols.

Subgroup analyses revealed some variability across age and sex groups, with slightly higher performance in younger patients and males, and modestly lower performance in older adults and certain female subgroups. These differences are likely related to a higher prevalence of ECG abnormalities, such as QRS and ST-T changes, bundle branch blocks, arrhythmias, electrolyte disturbances, and medication effects. Despite this variability, all AUROCs remained above 0.75,^39^ highlighting the model’s robustness across diverse patient subgroups. Complementary metrics, including precision-recall curves and F1-scores, confirmed the model’s robustness across demographics. Incorporating age and sex as inputs improved performance in certain subgroups, though future work may benefit from age-stratified or demographically adaptive architectures to better capture these nuances.

The model maintained diagnostic utility even under low-prevalence conditions, with AMI prevalence ranging from 5.9% to 9.6% across sites and a pooled AUPRC of approximately 0.45, well above chance. At the Youden-optimal threshold, sensitivity and specificity were balanced (73–78% and 65–80%, respectively), supporting its potential role in triage and prioritization. At a lower threshold optimized for NPV, the model achieved near-perfect sensitivity (>99%), highlighting its utility as a rule-out tool in emergency settings where missing AMI carries high clinical risk.

Interpretability remains a critical requirement for clinical adoption of AI systems. To address this, we generated SCORE-CAM maps, which consistently highlighted physiologically relevant ECG regions; the ST segment and T wave, which are known markers of MI.^6^ While attention-based methods do not fully explain a model’s decision process, this alignment with known pathophysiology lends support to the clinical plausibility of the predictions. Nevertheless, such interpretability techniques should be interpreted cautiously, and future studies may incorporate more rigorous feature attribution methods to enhance explainability.

To contextualize our model’s performance, we benchmarked it against several existing DL approaches, a ResNet-based model; originally trained on the PTB Diagnostic ECG Database^30^ for MI detection, a large-scale pretrained model from the CODE study,^14^ and the recently proposed ECG-SMART-NET.^15^ While these models performed well on curated datasets, their accuracy was lower when applied to our cohort. In comparison, the proposed model maintained relatively consistent performance across all sites, highlighting the importance of representative training data and model design that accommodates diverse emergency department populations. These findings suggest that generalizability to real-world settings depends on both the diversity of training data and thoughtful architectural choices tailored to the clinical context.

Despite the strengths of this study, including a large multi-centre cohort and rigorous cross-site validation, several limitations merit consideration. Outcome labelling relied on ICD codes, which may under-detect some AMI cases due to under-coding by clinicians. While these codes are assigned by trained professional staff and are generally reliable for this pilot study, the registry does not distinguish STEMI from NSTEMI/OMI, preventing subtype-specific analyses and potentially affecting model performance estimates. All hospitals were located in Sweden, resulting in geographic and demographic homogeneity that may limit broader applicability. Performance also varied across hospitals, likely reflecting differences in cohort size, prevalence, and heterogeneity of ECG acquisition and pre-processing. Low AMI prevalence introduced class imbalance, and subgroup analyses revealed age-related disparities, highlighting the potential benefits of demographically adaptive modelling. The retrospective design and limited structured symptom data (∼4%) may have introduced labelling biases. Finally, standardized ECG pre-processing could further improve model robustness.

This model is not intended to replace clinician judgment or laboratory diagnostics, such as cardiac biomarkers. Rather, its greatest value may lie in augmenting early triage by flagging high-risk ECGs for expedited review and supporting rule-out decisions in low-risk cases. To fully understand its clinical impact, prospective studies are needed to evaluate effects on diagnostic accuracy, workflow efficiency, treatment timelines, and patient outcomes.

In conclusion, this study provides compelling evidence that a carefully designed and externally validated DL model can support early AMI detection from routine ECGs across diverse emergency care settings. Realizing its full potential will require prospective validation and real-world implementation studies to ensure safety, effectiveness, and equitable integration into clinical workflows.

## Supplementary Material

ztaf125_Supplementary_Data

## Data Availability

Data cannot be shared/public due to ethical and privacy considerations. It includes sensitive patient information and disease diagnoses specific to the hospital setting.
