# Supplementary material for: Multimodal deep learning for acute myocardial infarction detection from 12-lead electrocardiogram: a multi-centre study with cross-hospital validation
Source: Eur Heart J Digit Health. 2025 Oct 27;7(2):ztaf125. doi: 10.1093/ehjdh/ztaf125 (PMC12853118; doi:10.1093/ehjdh/ztaf125)
Supplement: ztaf125_Supplementary_Data [file ztaf125_supplementary_data.docx]

**Supplementary Figures**


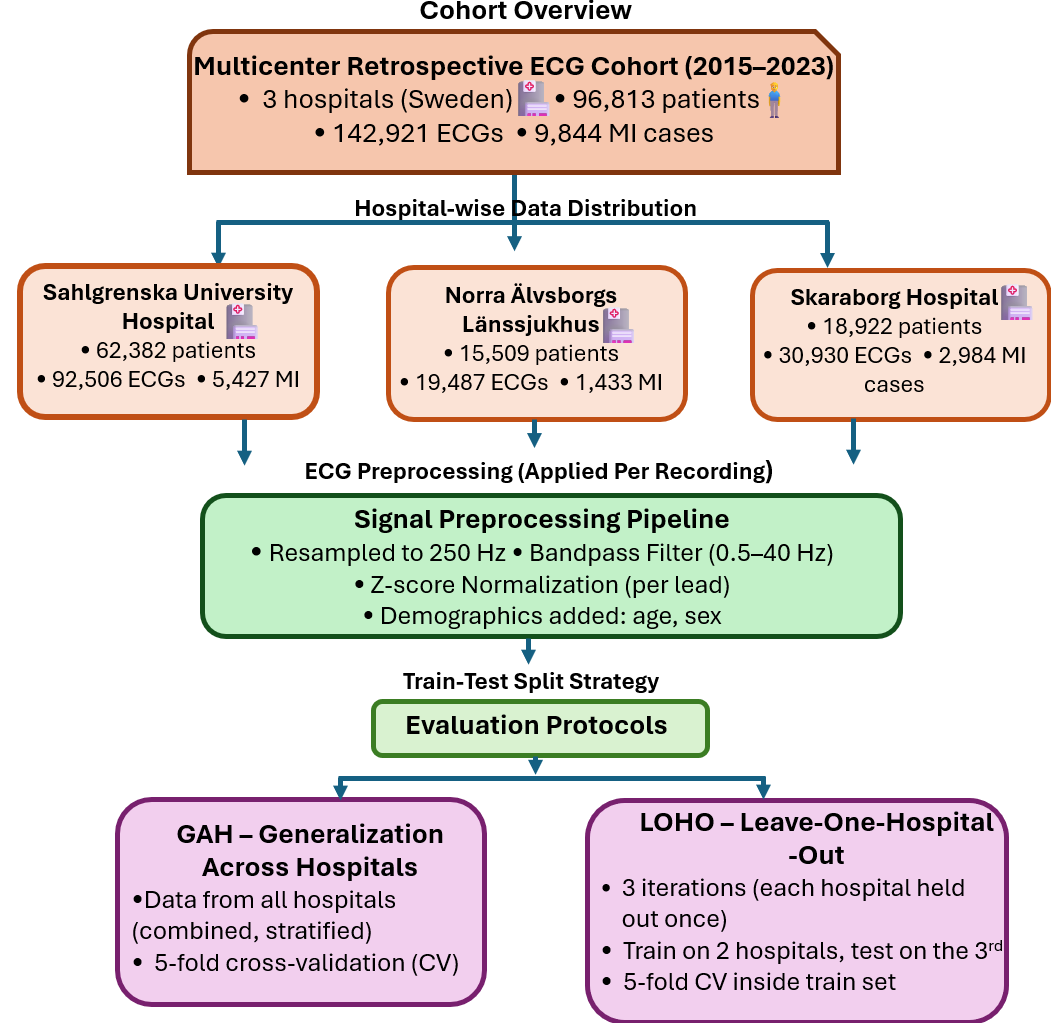


**Supplementary Figure 1. Study Overview and Data Processing Pipeline:** A schematic of study population, hospital-wise data contribution, ECG preprocessing, and model evaluation protocols (GAH and LOHO).


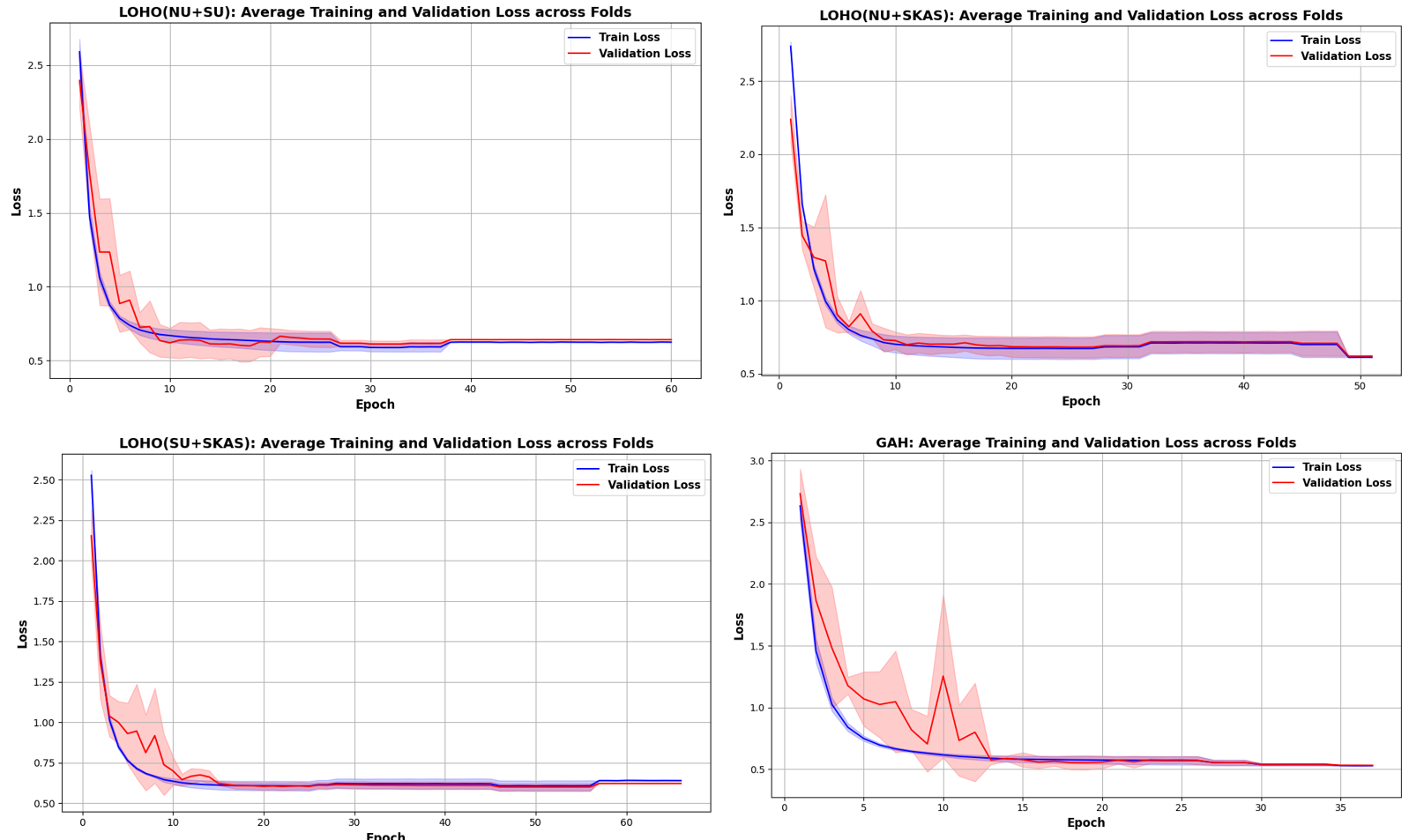


**Supplementary Figure 2. Training and validation loss curves**. Loss trends over training epochs for cross-validation folds.

**
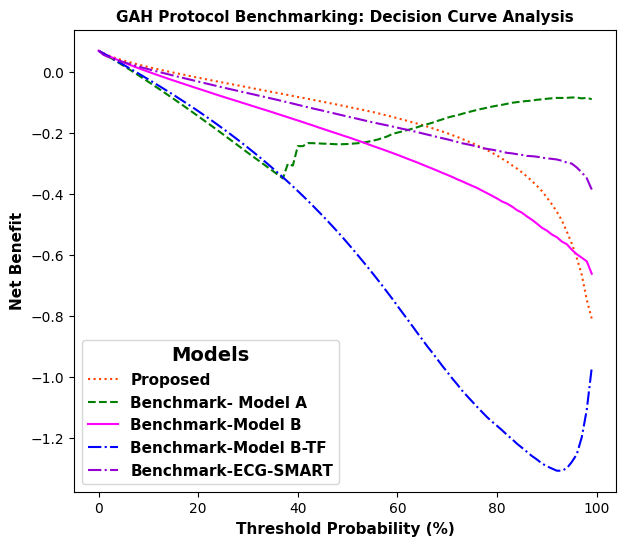
**

**Supplementary Figure 3. Decision curve analysis.** Clinical net benefit curves comparing the Conv-BiLSTM-Attn model and benchmark models, across varying decision thresholds.

**
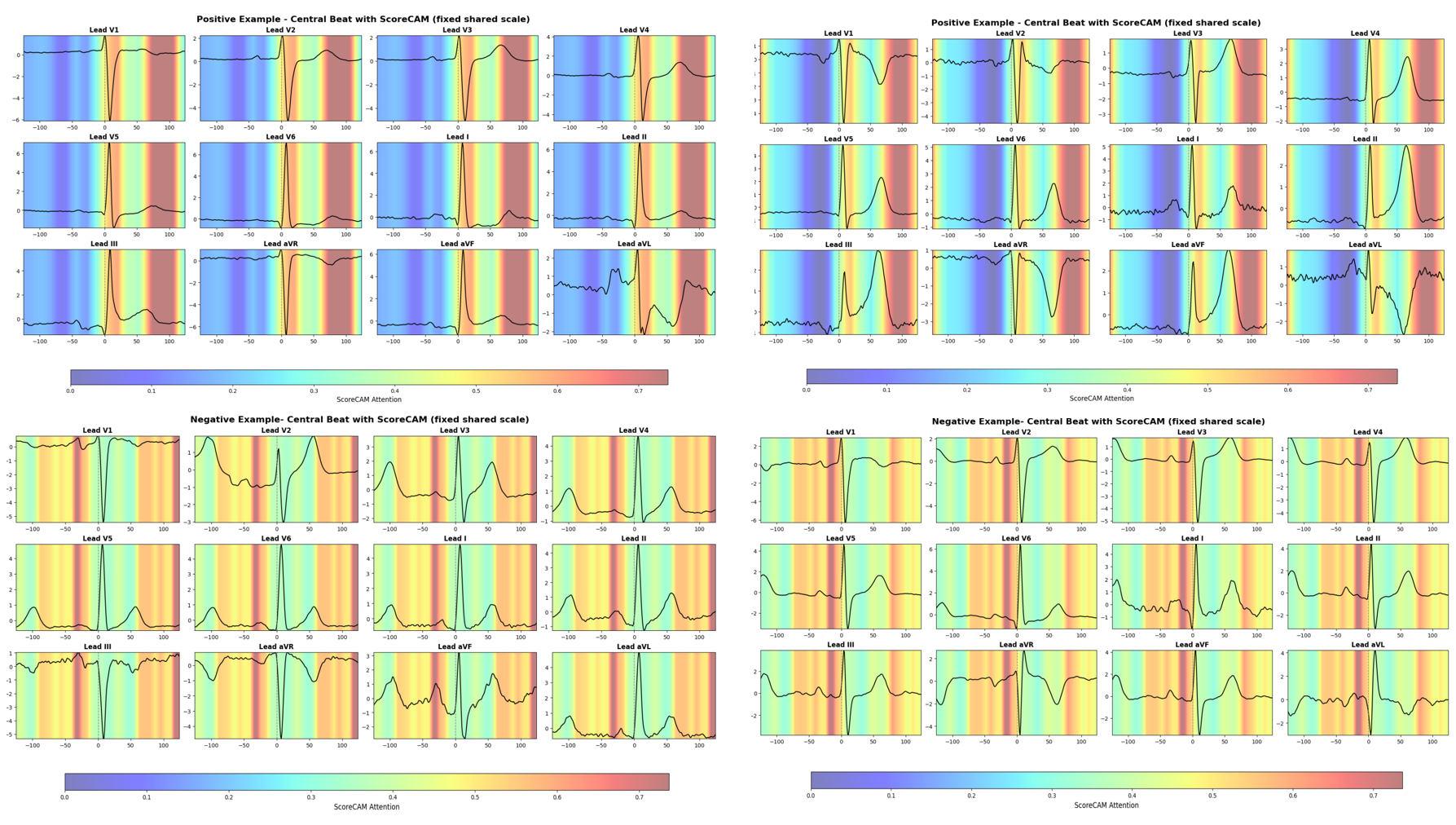
**

**Supplementary Figure 4.** Representative individual ScoreCAM attention maps plotted with a fixed global scale (0–95th percentile of all attention values), provided to complement Figure 5. (Top row) Two correctly classified AMI-positive cases; (bottom row) two correctly classified AMI-negative cases. Warmer colours (yellow/red) indicate stronger model attention, while cooler colours (blue) indicate less relevant regions. These examples, shown with a consistent fixed scale, confirm that the model highlights physiologically relevant ECG regions in AMI-positive patients, whereas attention is more diffuse in AMI-negative cases. Different representative cases (not those in Figure 5) are shown to demonstrate patient-level consistency.

**Supplementary Tables**

**Supplementary Table 1.** Threshold-specific diagnostic performance under LOHO and GAH protocols. For LOHO, results are reported separately for each hospital (NU, SU, SKAS). Metrics are shown at the Youden-optimal threshold and a fixed low threshold of 0.01, selected to prioritize high NPV. Reported values include sensitivity, specificity, NPV, binary and weighted F1 scores, and the corresponding Youden threshold, with 95% confidence intervals.

| **Metric** | **NU Hospital (LOHO)** | **SU Hospital (LOHO)** | **SKAS Hospital (LOHO)** | **GAH (Pooled)** |
| --- | --- | --- | --- | --- |
| **Youden-Optimal Threshold** | | | | |
| Sensitivity | 0.737 (0.716–0.773) | 0.671 (0.636–0.709) | 0.776 (0.744–0.805) | 0.736 (0.714–0.764) |
| Specificity | 0.789 (0.745–0.809) | 0.801 (0.770–0.822) | 0.651 (0.611–0.697) | 0.793 (0.764–0.812) |
| NPV | 0.974 (0.973–0.976) | 0.975 (0.973–0.977) | 0.965 (0.962–0.967) | 0.976 (0.974–0.978) |
| F1 Score (Binary) | 0.337 (0.311–0.348) | 0.276 (0.263–0.290) | 0.308 (0.294–0.325) | 0.325 (0.308–0.338) |
| F1 Score (Weighted) | 0.832 (0.806–0.844) | 0.844 (0.826–0.856) | 0.731 (0.705–0.762) | 0.837 (0.820–0.848) |
| Youden Threshold | 0.452 (0.362–0.480) | 0.338 (0.291–0.408) | 0.500 (0.500–0.500) | 0.439 (0.380–0.480) |
| **Fixed Threshold = 0.01** | | | | |
| Sensitivity | 0.995 (0.990–0.998) | 0.990 (0.964–1.000) | 0.996 (0.992–0.999) | 0.994 (0.992–0.996) |
| Specificity | 0.167 (0.141–0.229) | 0.133 (0.010–0.273) | 0.055 (0.024–0.094) | 0.168 (0.166–0.170) |
| NPV | 0.998 (0.997–0.999) | 0.998 (0.992–0.999) | 0.994 (0.991–0.997) | 0.997 (0.997–0.998) |
| F1 Score (Binary) | 0.160 (0.155–0.169) | 0.125 (0.112–0.142) | 0.184 (0.179–0.189) | 0.150 (0.147–0.153) |
| F1 Score (Weighted) | 0.276 (0.240–0.357) | 0.217 (0.024–0.410) | 0.110 (0.060–0.173) | 0.278 (0.275–0.281) |

**Supplementary Table 2.** Detailed architecture and training hyperparameters of the proposed model. The table includes input shapes, convolutional and recurrent layer configurations, attention and fully connected layers, output specifications, loss function, optimizer settings, training callbacks, and number of epochs.

| \| **Component** \| **Layer Type / Details** \| **Parameters / Notes** \| \| --- \| --- \| --- \| \| **Input** \| ECG input \| Shape: (2500, 12) \| \|  \| Age input \| Shape: (1,) \| \|  \| Sex input \| Shape: (1,) \| \| **CNN Feature Extractor** \| Conv1D \| 128 filters, kernel=15, strides=2, L2=0.0005 \| \|  \| BatchNorm + ReLU \| - \| \|  \| MaxPool1D \| pool=2, stride=2 \| \|  \| Conv1D \| 256 filters, kernel=9, L2=0.0005 \| \|  \| BatchNorm + ReLU \| - \| \|  \| MaxPool1D \| pool=2, stride=2 \| \| **Dilated Residual Block** \| Conv1D \| 256 filters, kernel=5, dilation=2, L2=0.0005 \| \|  \| BatchNorm + ReLU \| - \| \|  \| Conv1D \| 256 filters, kernel=3, L2=0.0005 \| \|  \| BatchNorm \| - \| \|  \| Add & ReLU \| Residual connection \| \| **Additional Conv Layers** \| Conv1D \| 256 filters, kernel=3, L2=0.0005 \| \|  \| BatchNorm + ReLU \| - \| \|  \| Conv1D \| 128 filters, kernel=3, L2=0.0005 \| \|  \| BatchNorm + ReLU \| MaxPool1D pool=2, stride=2 \| \| **BiLSTM** \| Bidirectional LSTM \| 128 units, return_sequences=True, dropout=0.2, recurrent_dropout=0.2 \| \|  \| Bidirectional LSTM \| 64 units, return_sequences=True, dropout=0.2, recurrent_dropout=0.2 \| \| **Attention** \| Attention \| GlobalAveragePooling1D applied \| \| **Fully Connected** \| Dense \| 256 units, ReLU, L2=0.0005, Dropout=0.3 \| \|  \| Dense \| 128 units, ReLU, L2=0.0005, Dropout=0.3 \| \|  \| Concatenate \| With age and sex inputs \| \|  \| Dense \| 64 units, ReLU, L2=0.0005, Dropout=0.2 \| \| **Output** \| Dense \| 2 units, Softmax \| \| **Loss Function** \| Binary Crossentropy \| Label smoothing=0.1 \| \| **Optimizer** \| Adam \| Learning rate=0.0001 \| \| **Metrics** \| Accuracy, Recall, Precision, AUC \| - \| \| **Training Callbacks** \| ReduceLROnPlateau \| Monitor='val_AUC', factor=0.1, patience=3 \| \|  \| EarlyStopping \| Monitor='val_loss', patience=8 \| \|  \| ModelCheckpoint \| Save best and last model \| \| **Epochs** \| - \| 100 \| \|  \|  \|  \| |
| --- | --- | --- | --- | --- | --- | --- | --- | --- | --- | --- | --- | --- | --- | --- | --- | --- | --- | --- | --- | --- | --- | --- | --- | --- | --- | --- | --- | --- | --- | --- | --- | --- | --- | --- | --- | --- | --- | --- | --- | --- | --- | --- | --- | --- | --- | --- | --- | --- | --- | --- | --- | --- | --- | --- | --- | --- | --- | --- | --- | --- | --- | --- | --- | --- | --- | --- | --- | --- | --- | --- | --- | --- | --- | --- | --- | --- | --- | --- | --- | --- | --- | --- | --- | --- | --- | --- | --- | --- | --- | --- | --- | --- | --- | --- | --- | --- | --- | --- | --- | --- | --- | --- | --- | --- | --- |

**Supplementary Table 3.** Summary of comorbidities at hospital admission. The table shows the most common comorbidities for each hospital, with the absolute number of patients and the percentage of the hospital population affected.

| **Center** | **ICD-10 Code** | **Patients with Code** | **Total Patients** | **% of Hospital Patients** | **Comorbidity** |
| --- | --- | --- | --- | --- | --- |
| NU | I109 | 2735 | 19487 | 14.03 | Hypertension |
| NU | I509 | 1399 | 19487 | 7.18 | Heart Failure |
| NU | I251 | 1222 | 19487 | 6.27 | Chronic Ischemic Heart Disease |
| NU | R074 | 1159 | 19487 | 5.95 | Chest Pain |
| NU | I489 | 1002 | 19487 | 5.14 | Atrial Fibrillation |
| NU | I252 | 910 | 19487 | 4.67 | Old Myocardial Infarction |
| NU | E119 | 907 | 19487 | 4.65 | Diabetes Mellitus (Type 2, non-insulin dependent) |
| NU | Z921 | 712 | 19487 | 3.65 | History of Medication Use |
| NU | Z955 | 585 | 19487 | 3.00 | Presence of Cardiac Device |
| NU | E785 | 562 | 19487 | 2.88 | Hyperlipidemia |
| SKAS | I109 | 11125 | 30929 | 35.97 | Hypertension |
| SKAS | I509 | 5338 | 30929 | 17.26 | Heart Failure |
| SKAS | R074 | 4988 | 30929 | 16.13 | Chest Pain |
| SKAS | I489 | 4475 | 30929 | 14.47 | Atrial Fibrillation |
| SKAS | E119 | 4465 | 30929 | 14.44 | Diabetes Mellitus (Type 2, non-insulin dependent) |
| SKAS | I252 | 3254 | 30929 | 10.52 | Old Myocardial Infarction |
| SKAS | Z921 | 3180 | 30929 | 10.28 | History of Medication Use |
| SKAS | J449 | 2714 | 30929 | 8.77 | Chronic Obstructive Pulmonary Disease (COPD) |
| SKAS | Z955 | 2139 | 30929 | 6.92 | Presence of Cardiac Device |
| SKAS | J441 | 2091 | 30929 | 6.76 | Chronic Bronchitis |
| SU | I109 | 12958 | 92505 | 14.01 | Hypertension |
| SU | R074 | 6375 | 92505 | 6.89 | Chest Pain |
| SU | I509 | 6349 | 92505 | 6.86 | Heart Failure |
| SU | I489 | 5212 | 92505 | 5.63 | Atrial Fibrillation |
| SU | E119 | 4906 | 92505 | 5.30 | Diabetes Mellitus (Type 2, non-insulin dependent) |
| SU | I259 | 4030 | 92505 | 4.36 | Chronic Ischemic Heart Disease (unspecified) |
| SU | I251 | 2881 | 92505 | 3.11 | Chronic Ischemic Heart Disease |
| SU | E785 | 2676 | 92505 | 2.89 | Hyperlipidemia |
| SU | I252 | 2620 | 92505 | 2.83 | Old Myocardial Infarction |
| SU | J449 | 2543 | 92505 | 2.75 | Chronic Obstructive Pulmonary Disease (COPD) |
